# Supplementary material for: MetaRibo-Seq measures translation in microbiomes
Source: Nat Commun. 2020 Jun 29;11:3268. doi: 10.1038/s41467-020-17081-z (PMC7324362; doi:10.1038/s41467-020-17081-z)
Supplement: Supplementary file 10 — Supplementary Data 7 [file 41467_2020_17081_MOESM10_ESM.zip › File2/Confidence_High_Taxonomy/132377_out.krona.html]

Javascript must be enabled to view this page.

members
magnitude
magnitudeUnassigned
count
unassigned
taxon
rank

132377\_out

147

2
superkingdom
147

201174
phylum
1

class
1
1760

order
1
85006

1268
1
family

1
genus
32207

172042

SRS050783\_contig\_number\_57
1
species

1239
2
phylum

909932
class
2

1
order
909929

1843491
1
family


SRS013098\_contig\_number\_18041
158846
genus
1

1843488
order
1

1
family
909930

1
genus
33024

species
1
626940

SRS049959\_contig\_number\_13365

1224
144
phylum

28216
class
92

88
order
80840

995019
33
family

1980697
4
genus

species
4

SRS1041037\_contig\_number\_599SRS893230\_contig\_number\_199SRS893230\_contig\_number\_contig-100\_547.35349SRS893327\_contig\_number\_190
1852381

genus

SRS013940\_contig\_number\_contig-100\_1414.1415SRS098585\_contig\_number\_contig-100\_2317.2317SRS143181\_contig\_number\_contig-100\_10133.77231
40544
29
3

24
species
40545

SRS014415\_contig\_number\_contig-100\_9418.9419SRS014948\_contig\_number\_13135SRS016095\_contig\_number\_1420SRS016989\_contig\_number\_210SRS017247\_contig\_number\_2253SRS017433\_contig\_number\_1109SRS022609\_contig\_number\_13166SRS024331\_contig\_number\_4133SRS024549\_contig\_number\_contig-100\_17.17SRS043667\_contig\_number\_contig-100\_1041.1041SRS047433\_contig\_number\_1563SRS050026\_contig\_number\_992SRS050520\_contig\_number\_144SRS052697\_contig\_number\_11970SRS057717\_contig\_number\_887SRS063370\_contig\_number\_8491SRS063370\_contig\_number\_contig-100\_193.194SRS064232\_contig\_number\_925SRS075878\_contig\_number\_1124SRS077086\_contig\_number\_198SRS1041039\_contig\_number\_20573SRS1041138\_contig\_number\_540SRS1054716\_contig\_number\_9302SRS147653\_contig\_number\_348


SRS1055022\_contig\_number\_345
1262975
1
species


SRS142781\_contig\_number\_287
2292254
1
species

family
3
80864

219181
3
genus

1
species
1658672

SRS043018\_contig\_number\_4

2
species
2045302

SRS021477\_contig\_number\_269SRS063603\_contig\_number\_165

119060
family
52

52
genus
47670

2
species

SRS051786\_contig\_number\_contig-100\_1617.1617SRS893258\_contig\_number\_116
2490857

species
50

SRS012285\_contig\_number\_78SRS013945\_contig\_number\_58SRS014941\_contig\_number\_29SRS015989\_contig\_number\_189SRS018145\_contig\_number\_394SRS018691\_contig\_number\_144SRS019225\_contig\_number\_48SRS020340\_contig\_number\_8751SRS023964\_contig\_number\_993SRS042307\_contig\_number\_contig-100\_63.64SRS043729\_contig\_number\_51SRS045262\_contig\_number\_37SRS047126\_contig\_number\_272SRS047590\_contig\_number\_976SRS047634\_contig\_number\_39824SRS053895\_contig\_number\_2SRS055401\_contig\_number\_contig-100\_8.8SRS058075\_contig\_number\_234SRS062931\_contig\_number\_1SRS063672\_contig\_number\_contig-100\_391.392SRS063678\_contig\_number\_815SRS064937\_contig\_number\_729SRS075406\_contig\_number\_11SRS077635\_contig\_number\_contig-100\_292.293SRS077639\_contig\_number\_66SRS078003\_contig\_number\_11412SRS097918\_contig\_number\_contig-100\_1239.1240SRS098482\_contig\_number\_215SRS104045\_contig\_number\_contig-100\_512.513SRS104320\_contig\_number\_13107SRS104521\_contig\_number\_17SRS1055042\_contig\_number\_17SRS1055091\_contig\_number\_contig-100\_2304.28052SRS142608\_contig\_number\_271SRS143141\_contig\_number\_290SRS143996\_contig\_number\_contig-100\_204.204SRS144306\_contig\_number\_contig-100\_152.153SRS144703\_contig\_number\_178SRS144761\_contig\_number\_288SRS146874\_contig\_number\_344SRS146878\_contig\_number\_contig-100\_226.226SRS147076\_contig\_number\_contig-100\_134.135SRS148152\_contig\_number\_contig-100\_142.143SRS148520\_contig\_number\_54SRS148979\_contig\_number\_contig-100\_201.202SRS149770\_contig\_number\_130SRS893232\_contig\_number\_484SRS893233\_contig\_number\_66SRS893281\_contig\_number\_1819SRS893338\_contig\_number\_107
47671

4
order
206351

481
family
4

genus
2
482

species
1

SRS149184\_contig\_number\_698
1715112

607712

SRS104521\_contig\_number\_53
1
species

32257
genus
2

505

SRS050974\_contig\_number\_681SRS149973\_contig\_number\_contig-100\_152.152
species
2

class
52
1236

135615
4
order

868
family
4

4
genus
2717


SRS017634\_contig\_number\_196SRS147128\_contig\_number\_11894SRS893299\_contig\_number\_107SRS893338\_contig\_number\_contig-100\_10631.68053
2718
4
species

91347
order
2

family
2
543

genus
2
561

562

SRS143423\_contig\_number\_2616SRS144362\_contig\_number\_2281
2
species

135625
46
order

712
family
46

genus
21
416916

3
species
2025884

SRS013533\_contig\_number\_45032SRS062931\_contig\_number\_7688SRS1055086\_contig\_number\_729

species
9
739

SRS019029\_contig\_number\_1634SRS022883\_contig\_number\_1938SRS064260\_contig\_number\_11708SRS103983\_contig\_number\_14902SRS1041183\_contig\_number\_3351SRS104320\_contig\_number\_207SRS104539\_contig\_number\_24328SRS149237\_contig\_number\_425SRS893376\_contig\_number\_1

species
9

SRS018929\_contig\_number\_74SRS019798\_contig\_number\_contig-100\_650.651SRS024447\_contig\_number\_49SRS045605\_contig\_number\_contig-100\_8009.40551SRS055378\_contig\_number\_12178SRS065099\_contig\_number\_1SRS077639\_contig\_number\_contig-100\_425.33691SRS105121\_contig\_number\_142SRS148730\_contig\_number\_contig-100\_2484.36520
732


SRS018149\_contig\_number\_53
724
25
1
genus

1
species
1739452

SRS045262\_contig\_number\_contig-100\_81.81

712310

SRS018978\_contig\_number\_469SRS145505\_contig\_number\_contig-100\_4808.35100
species
2

726

SRS011144\_contig\_number\_76SRS042409\_contig\_number\_contig-100\_263.263SRS046686\_contig\_number\_859SRS077308\_contig\_number\_218SRS1055061\_contig\_number\_contig-100\_1203.1204SRS147670\_contig\_number\_310SRS148726\_contig\_number\_contig-100\_854.855SRS149282\_contig\_number\_434
8
species

1
species

SRS015062\_contig\_number\_contig-100\_452.452
727


SRS013506\_contig\_number\_contig-100\_2036.2036SRS019439\_contig\_number\_398SRS077241\_contig\_number\_contig-100\_126.127SRS1055080\_contig\_number\_1937
736
species
4


SRS045588\_contig\_number\_contig-100\_1248.1249SRS078419\_contig\_number\_142SRS104036\_contig\_number\_1825SRS145497\_contig\_number\_contig-100\_104.104
729
species
4


SRS014530\_contig\_number\_contig-100\_1781.1782SRS015061\_contig\_number\_contig-100\_954.954SRS075086\_contig\_number\_861SRS893301\_contig\_number\_5133
735
species
4
